# CRP-Cyclic AMP Dependent Inhibition of the Xylene-Responsive σ^54^-Promoter *Pu* in *Escherichia coli*

**DOI:** 10.1371/journal.pone.0086727

**Published:** 2014-01-23

**Authors:** Yuan-Tao Zhang, Feng Jiang, Zhe-Xian Tian, Yi-Xin Huo, Yi-Cheng Sun, Yi-Ping Wang

**Affiliations:** 1 State Key Laboratory of Protein and Plant Gene Research, College of Life Sciences, Peking University, Beijing, China; 2 Institute of Pathogen Biology, Chinese Academy of Medical Sciences and Peking Union Medical College, Beijing, China; Indian Institute of Science, India

## Abstract

The expression of σ^54^-dependent *Pseudomonas putida Pu* promoter is activated by XylR activator when cells are exposed to a variety of aromatic inducers. In this study, the transcriptional activation of the *P. putida Pu* promoter was recreated in the heterologous host *Escherichia coli*. Here we show that the cAMP receptor protein (CRP), a well-known carbon utilization regulator, had an inhibitory effect on the expression of *Pu* promoter in a cAMP-dependent manner. The inhibitory effect was not activator specific. *In vivo* KMnO_4_ and DMS footprinting analysis indicated that CRP-cAMP poised the RNA polymerase at *Pu* promoter, inhibiting the isomerization step of the transcription initiation even in the presence of an activator. Therefore, the presence of PTS-sugar, which eliminates cAMP, could activate the poised RNA polymerase at *Pu* promoter to transcribe. Moreover, the activation region 1 (AR1) of CRP, which interacts directly with the αCTD (C-terminal domain of α-subunit) of RNA polymerase, was found essential for the CRP-mediated inhibition at *Pu* promoter. A model for the above observations is discussed.

## Introduction

The σ^54^-dependent *Pu* promoter drives transcription of the *upper* operon of *Pseudomonas putida* mt-2 TOL plasmid pWW0 for degradation of toluene and xylenes [1]–[4]. This promoter region includes two upstream activating sites (UASs) for the activator protein XylR [5], [6], a −12/−24 region recognized by Eσ^54^ RNA polymerase, a single integration host factor (IHF) binding site located in the intervening region[5], [7] and the adjacent UP-like elements for docking of the Eσ^54^ [8]. IHF-mediated DNA bending facilitates the direct interactions between two CTDs of the RNA polymerase and two separated UP-like elements located −78 and −104 upstream of the transcriptional start site [8], recruiting Eσ^54^ to the *Pu* promoter when cells reach stationary phase [9], [10]. It is generally believed that this IHF-dependent closed complex formation is the rate-limiting step for the transcriptional initiation at the *Pu* promoter [8], [11].

Recently, the architectural organization of the σ^54^-dependent promoter was investigated and led to the conclusion that the activator must approach the Eσ^54^ closed complexes from the unbound (activator accessible) face of the promoter DNA helix to catalyze open complex formation [12]. This conclusion is further supported by the first modeling of activator-promoter DNA-Eσ^54^ complex [12]. Since the contact between the UAS bound activator and promoter bound Eσ^54^ depends on the orientation of the DNA bending between UAS and −12/−24 region of a promoter [13], [14], the optimal IHF induced DNA bending at *Pu* promoter is essential for the transcription initiation [8].

CRP, the cyclic AMP (cAMP) receptor protein, is one of the best studied transcriptional factor, which is responsible for the regulation of more than 100 genes mainly involved in catabolism of sugars, amino acids and nucleotides in *E.coli* [15], [16]. The CRP-mediated regulation requires initially the binding of cAMP to form an active CRP homodimer when intracellular cAMP level is high, but in the presence of PTS (phosphoenolpyruvate-sugar phosphotransferase system)-sugars such as glucose the low cAMP level diminishes the activity of CRP [16]. At the σ^70^-dependent promoters, the dimeric CRP protein enhances the ability of Eσ^70^ to bind DNA and initiate transcription by interacting with Eσ^70^ directly [17]. Two discrete surfaces of CRP, known as Activating Region 1 (AR1, consisting of residues 156–164 of CRP) and Activating Region 2 (AR2, consisting of residues His19, His21 and Lys101 of CRP), interact with the C-terminal and N-terminal domains, respectively, of the α subunit of RNA polymerase [15] and the structural basis of CRP-αCTD-DNA complex has been determined [18]. A third contact surface (AR3) of CRP which is thought to interact with σ^70^ only at Class II promoters has also been identified. AR3 is defined as containing both the activating (residues 53, 54, 55 and 58 of CRP) and inhibitory (residue 52 of CRP) determinants [19].

Previous studies showed that CRP-cAMP down-regulated the σ^54^-dependent *dctA* and *glnA*p2 promoters in *Escherichia coli* [20]–[22]. Two mechanisms are involved for the CRP-mediated inhibition of the expression of *glnA*p2 promoter [23]. First, CRP affects GlnB signaling through direct activation of *glnHPQ* operon and in turn de-activates *glnA*p2. Second, *in vitro* studies show that CRP can be recruited by Eσ^54^ to a site upstream of *glnA*p2 through the direct interaction between αCTD of Eσ^54^ and AR1 of CRP, preventing the activator protein from approaching the activator-accessible face of the promoter-bound Eσ^54^ closed complex [23]. Therefore, as the major transcriptional effector of the ‘glucose effect’, CRP affects both the signal transduction pathway and the overall geometry of the transcriptional machinery of components of the nitrogen regulon. As a result, *E.coli glnA*p2, *glnH*p2[23], *glnK* (unpublished observations) and *astABCDE* promoters [24] together with the *Klebsiella pneumoniae nifB*, *nifE*, *nifF*, *nifH*, *nifJ*, *nifLA* and *nifU* promoters [25] are all down-regulated by the CRP-cAMP complex in *E.coli*.

Based on the striking inhibitory effect of CRP-cAMP on the expression of σ^54^-dependent promoters as mentioned above, this study was undertaken to investigate the influence of CRP-cAMP on the *Pu* promoter expression in *E. coli*. Our data show that CRP had an inhibitory effect on the expression of *Pu* promoter in a cAMP-dependent manner and the inhibitory effect was not activator specific. *In vivo* KMnO_4_ and DMS footprinting analysis indicated that CRP-cAMP poised the RNA polymerase at *Pu* promoter, inhibiting the isomerization step of the transcription initiation even in the presence of an activator. This transcription program leads to the maximal production of toluene and xylenes degradation enzymes only in the absence of cAMP signal. Moreover, AR1 of CRP, which interacts directly with the αCTD of RNA polymerase, was found essential for this CRP-mediated inhibition at *Pu* promoter. A model for the above observations is discussed.

## Materials and Methods

### Bacterial strains and plasmids

Bacterial strains and plasmids used in this study are listed in Table 1.

**Table 1:** Bacterial strains and plasmids used in this work.

Strain/Plasmid	Relevant characteristics	Source/Reference
*E.coli* strains		
TP2101	F-, *xyl*, *lac*Δ*X74*, *argH1*	A. Danchin
TP2006	F-, *xyl*, *cya*, *lac*Δ*X74*, *argH1*, *glp* ^*^	[57]
TP2339	F-, *xyl*, *cya*, *crp-39*, *lac*Δ*X74*, *argH1*	[22]
TP2339-1	F-, *xyl*, *cya*, *crp-39*, *lac*Δ*X74*, *argH1*, *glp* ^*^	[22]
TH1	Δ*lacU169*, *endA1*, *thi-1*, *hsdR17*, *supE44*, Δ*rpoN2518*	
Plasmids		
pWW0	TOL^+^, IncP9 incompatibility group plasmid	Juan L. Ramos
pBluscript-SK	ColE1, *lacZ*', Ap^R^	Stratagene
pBS/*Pu*	pBluscript-SK::Pp *Pu*, Ap^R^	This work
pGD926	*lacZYA* translational fusion vector, Tc^R^	[29]
pKU101	*glnA*p2*mCRP::lacZYA* fusion in pGD926, Tc^R^	[22]
pKU700	*Pu::lacZYA* fusion in pGD926, Tc^R^	This work
pTS174	pACYC184 derivative, expresses *xylR*, Cm^R^	[31]
pVTRΔA	pVTR-A derivative, expresses *xylR*Δ*A*, Cm^R^	V. De Lorenzo
pLG339CRP	pLG339 carrying *E.coli crp* under the control of the *crp* promoter, Km^R^	[33]
pLG339PpCRP	pLG339 carrying *P.putida crp* under the control of constitutive Tc promoter, Km^R^	This work
pLG339ΔRS	pLG339 with EcoRI/SalI internal deletion, Km^R^	S. Busby
pLG339CRP H159L	pLG339CRP derivative, Km^R^, CRP with defective AR1	S. Busby
pLG339CRP K101E	pLG339CRP derivative, Km^R^, CRP with defective AR2	S. Busby
pLG339CRP K52N	pLG339CRP derivative, Km^R^, CRP with improved AR3	S. Busby
pLG339CRP E58K	pLG339CRP derivative, Km^R^, CRP with defective AR3	S. Busby

Ap, ampicillin; Cm, chloramphenicol; Km, kanamycin; Tc, tetracycline; ^R^, resistance; Pp, *Pseudomonas putida*; Δ, deletion; :s, novel joint; *lac*Δ*X74*, complete deletion of the *lac* operon; *glp* ^*^ is mutation near the *argH* gene that allows growth of this strain on glycerol [57].

### Growth media and enzyme assays

M63 modified medium was prepared as previously described [26]. Cells were grown at 30°C. β-galactosidase assays were performed according to Miller [27].

### Genetic manipulations

Preparation of plasmid DNA, restriction enzyme digestions, ligations and horizontal agarose gel electrophoresis in Tris-borate-EDTA buffer were performed according to the standard methods [28]. DNA sequence analysis was either performed at TaKaRa Corporation, Japan or using a GenomyxLR™-OPTIMIZED sequencing kit for DMS and KMnO_4_ footprinting experiment.

### Plasmid construction

The complete nucleotide sequence of *xyl* upper operon of TOL plasmid pWW0 was sequenced previously (Harayama *et al*., unpublished). In order to amplify the *Pu* promoter and its upstream sequence, two primers were synthesized: 5′-CCCAAGCTTAGCGCGATGAACCTTTTTTATCGC-3′ (p1, *Hin*dIII) and 5′- CGGGATCCGAGTTGAGAAAATACAACATTG-3′ (p2, *Bam*HI). Restriction sites present in oligonucleotide primers used for cloning are underlined. Polymerase Chain Reactions (PCRs) were carried out, and the entire *Pu* region and the first 7 codons of the *xylU* open reading frame (ORF) (from –200 to +50 of *xylU*) together with artificially introduced restriction sites was amplified, using TOL plasmid pWW0 from *P. putida* strain mt-2 as template and p1, p2 as primers. The DNA fragment was restricted with *Hin*dIII and *Bam*HI, and cloned into pBluescript-SK. This resulted in pBS/*Pu* and its DNA sequence was verified. The 250 bp *Hin*dIII-*Bam*HI fragment of pBS/*Pu* was subsequently inserted into pGD926 to produce an in frame *Pu*::*lac*Z fusion, plasmid pKU700. In order to amplify the *P. putida crp* gene, PCR was carried out by using *P. putida* strain mt-2 genome as template and oligonucleotides p3 (5′-CGCGGATCCTCACCGGCCCACTGGATACG, *Bam*HI) and p4 (5′-ACGCGTCGACCTAGCGGGTACCGTGGACC, *Sal*I) as primers. The *Bam*HI-*Sal*I fragment was verified subsequently and inserted into pLG339 to gain the plasmid designated as pLG339PpCRP.

### *In vivo* KMnO4 footprinting experiments

To detect Eσ^54^-DNA open complex, *E. coli* strain carrying the indicated plasmids was pre-grown aerobically at 30°C to late-logarithmic phase in the LB medium, diluted into 10 mL of the same medium with an addition of 0.2 mmol/L m-methylbenzyl alcohol (mMBA) as the inducer at an initial OD_600_ of 0.05 and then grown out aerobically. At 0.9 OD_600_, each sample was treated with 40 µL of 50 mg mL^−1^ rifampicin (dissolved in methanol) for 5 min. The cells were immediately spun down, followed by resuspension in 5 mL of 0.09 mol/L KMnO_4_ for 2 min. The reaction was stopped by adding 100 µL of β-mercaptoethanol. The cells were spun down, and the plasmid DNA was isolated using SV DNA purification kit (Promega Corporation). 7 µL of 100 µL eluted plasmid DNA was analyzed by PCR amplified primer extension. 7 µL of DNA solution, 2 µL of 5× Taq polymerase buffer, 1.5 µL dNTP (2.5 mmol/L each), 0.25 µL of 5′-^32^P-labeled p3 (0.4 pmol µL^−1^, 7.4×10^4^ Bq µL^−1^) (p3: 5′-GGGATGTGCTGCAAGGCGAT-3′, which hybridizes with the structural gene of *lacZ*) and 0.25 µL of sequencing grade Taq polymerase (5 U µL^−1^, Promega Corporation) were mixed together and denatured at 94°C for 1 min, hybridized at 47°C for 30 sec, and extended 30 sec at 72°C. This cycle was repeated 35 times. The samples were analyzed on a 6% (w/v) polyacrylamide sequencing gel, calibrated with the corresponding sequencing reactions.

### *In vivo* DMS footprinting experiments

To detect binding of Eσ^54^ RNA polymerase to *Pu* promoter, *E. coli* strain carrying the indicated plasmids was grown aerobically at 30°C in 50 mL LB medium, supplemented with 40 mmol/L NH_4_^+^ (for the *rpoN* mutant, additionally supplemented with 0.2% glutamine), in the presence or absence of 2 mmol/L exogenous cAMP. During the late-logarithmic growth, DMS was added (final concentration of 0.1% [w/v]) from a fresh 2% (w/v) solution in saline phosphate (SP) buffer. Cells were incubated for 1 min, then spun down and washed twice with 50 mL SP buffer. The plasmid DNA was isolated using SV DNA purification kit (Promega Corporation). 75 µL of 100 µL eluted plasmid DNA was cleaved with 10% (v/v) piperidine at 90°C for 30 min. Following piperidine cleavage they are purified through a 1 mL Sephadex G-50 spin DNA column (Roche Diagnostics Corporation). 35 µL of DNA in distilled water, 1 µL of 5′-^32^P-labeled p3 (0.4 pmol µL^−1^, 7.4×10^4^ Bq µL^−1^, for the sequence of p3, see *in vivo* KMnO_4_ footprinting experiments) for pKU700 or p4 (0.4 pmol µL^−1^, 7.4×10^4^ Bq µL^−1^) (p4: 5′-GGTTTTCCCAGTCACGACGTTG-3′) for pKU101 containing a *glnA*p2::*lacZ* fusion in the reporter plasmid pGD926[22] and 5 µL 0.01 mol NaOH were mixed well and then heated for 2 min at 90°C, followed plunged into ice. Then 8 µL of dNTP (2.5 mmol each), 10 µL of 10×EcoPol buffer, 41 µL of ddH_2_O, 0.2 µL of Klenow DNA polymerase (5 U µL^−1^, New England Biolabs, Inc) was added and extended at 37°C for 45 min. The samples were analyzed on a 6% (w/v) polyacrylamide sequencing gel, calibrated with the corresponding sequencing reactions.

## Results

### Differential induction of the *Pu* promoter in *E. coli cya* mutant TP2006 and its isogenic wild type TP2101

**Figure 1:**
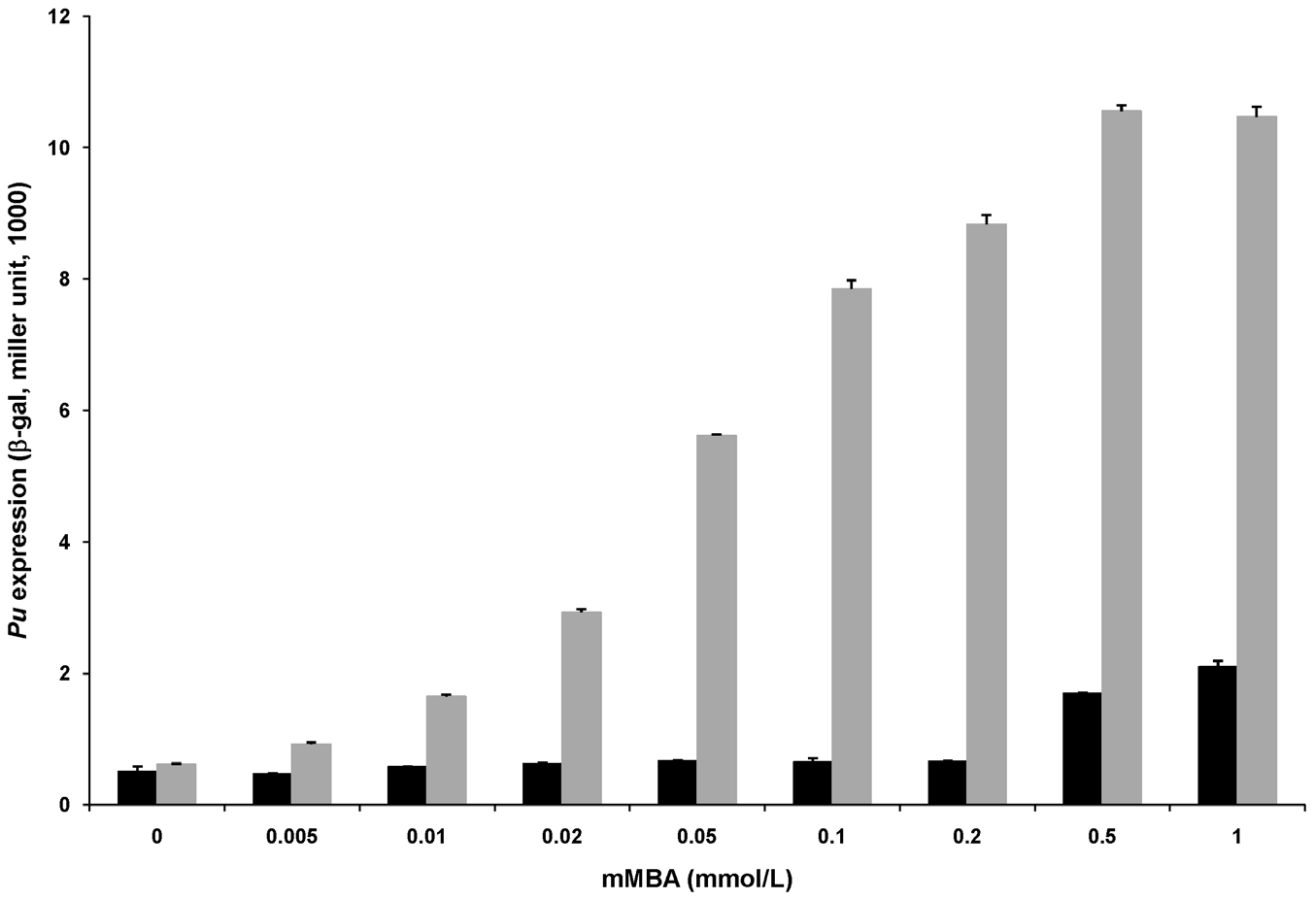
Influence of the quantity of mMBA on *Pu* expression in *E. coli*. *E. coli* wild type strain TP2101 and *cya* mutant TP2006 were co-transformed with pKU700 and pTS174. Transformants were grown in LB medium supplied with the increasing concentrations of mMBA as the inducer and then measured the β-galactosidase activities. The results are the mean of at least three independent experiments and include the standard deviation. Note the different induction profile of *Pu* promoter in TP2101 (black bars) and TP2006 (gray bars).

In this study, XylR-mediated activation of the *P. putida Pu* promoter was recreated in the heterologous host *E. coli*. A translational *Pu*::*lacZ* fusion was constructed in the 28 kb low-copy reporter plasmid pGD926 [29], a derivative of the Rk2, the best-studied IncP-1 plasmid whose replication and, hence, its copy number are tightly regulated [30]. This resulting construct was named pKU700. Plasmid pTS174, encoding constitutively expressed *P. putida* XylR activator [31] was used to activate the *Pu* promoter in *E. coli*. Plasmid pKU700 was co-transformed with pTS174 into *E. coli cya* mutant TP2006 (unable to produce cAMP) and its isogenic wild type TP2101 separately. β-galactosidase activities were measured in the presence of different concentrations of mMBA, an effective aromatic inducer for XylR [32]. Activator XylR-mediated and inducer mMBA-dependent activation of the *Pu* promoter was observed in both *E. coli* strains (Figure 1). However, under the same mMBA concentration, XylR-activated expression of *Pu* was much higher in the *cya* mutant TP2006 than that in its isogenic wild type strain TP2101 (Figure 1). In addition, control experiment by using empty vector of pTS174 showed little *Pu* activation in both *E. coli* strains (data not shown). These results suggest that the presence of cAMP affects *Pu* expression. Hence, it was thought necessary to examine whether the CRP-cAMP complex influenced *Pu* expression.

### CRP-cAMP-mediated conditional inhibition of the *Pu* promoter in *E. coli*

To explore the role of CRP-cAMP in controlling *Pu* output in *E. coli*, promoter activity was monitored in the *E. coli* wild-type strain TP2101, the *cya* mutant TP2006 and the *cya crp* double mutant TP2339-1 respectively. All strains harbor plasmids pTS174 and pKU700. In addition, low copy pLG339-derived plasmids [33], harboring either the wild-type *E. coli crp* gene (pLG339CRP), *P. putida* (pLG339PpCRP), or a deleted *crp* gene (pLG339ΔRS, empty vector), were introduced into TP2339-1. Expression of *Pu* was monitored in the absence or presence of cAMP. In the absence of cAMP, the *Pu* promoter was expressed at high levels in TP2006 and TP2339-1 (i.e., in hosts lacking cAMP) (Table 2). In contrast, it was inhibited in the wild-type strain TP2101 (Table 2). When exogenous cAMP was added in the growth medium, expression of *Pu* was comparatively lower than that in the absence of cAMP in TP2006 and TP2339-1 containing pLG339CRP (carrying the *E.coli crp* gene) or pLG339PpCRP (carrying the *P. putida crp* gene), but it remained constant at a high level in TP2339-1 containing pLG339ΔRS (Table 2). These results suggested that CRP proteins (both from *E. coli* and *P. putida*) had a cAMP-dependent inhibitory effect on *Pu* expression.

**Table 2:** The cAMP receptor protein (CRP) is the factor that mediates the inhibition of the *Pu* promoter in *E. coli*.

Strain	Plasmid	β-Gal activity (Miller units)*	
		Exogenous cAMP†	
		−	+
TP2101 (WT)	pKU700 + pTS174	223±35	217±30
TP2006 (*cya*)	pKU700 + pTS174	8837±352	576±45
TP2339-1(*cya crp*)	pKU700 + pTS174 + pLG339ΔRS	7953±307	8201±326
TP2339-1(*cya crp*)	pKU700 + pTS174 + pLG339CRP	7900±298	238±28
TP2339-1(*cya crp*)	pKU700 + pTS174 + pLG339PpCRP	5693±269	2983±334

* The β-galactosidase activity in LB medium with an addition of 0.2 mmol/L mMBA as the inducer was assayed after growing cells at 30°C. Mean values and standard deviations from three independent experiments are shown.

† Exogenous cAMP was at a final concentration of 2 mmol/L when added.

### CRP-cAMP-mediated inhibition on *Pu* is at the transcriptional level *in vivo*

**Figure 2:**
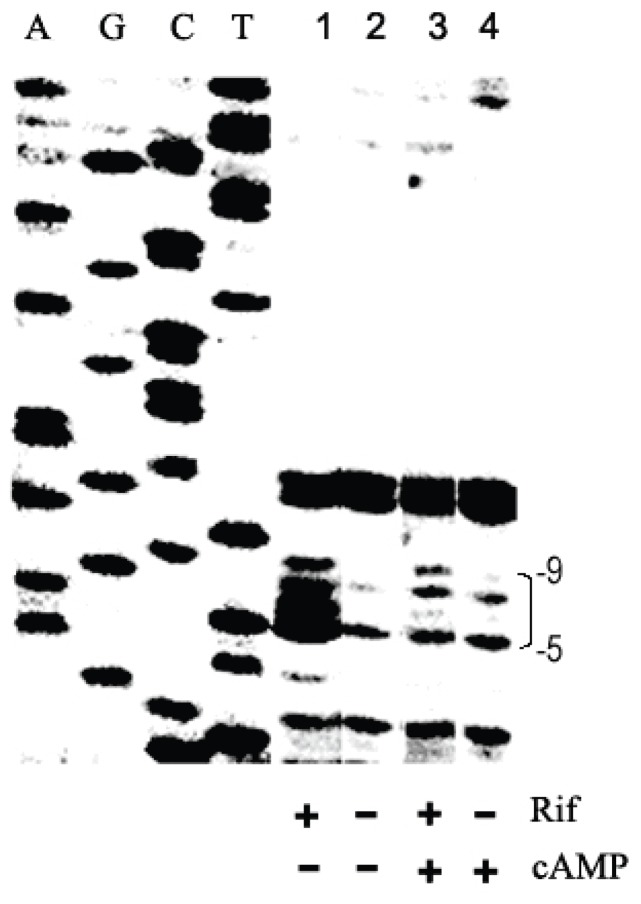
Probing open complexes at the *Pu* promoter with KMnO_4_. Under activated situation, KMnO_4_ footprints were conducted on plasmid pKU700 in *E. coli cya* mutant TP2006. Lanes: 1, plus rifampicin; 2, no rifampicin; 3, plus rifampicin and 2 mmol/L cAMP; 4, plus 2 mmol/L cAMP; A, G, C and T refer to sequencing lanes with the same primer. The −5 to −9 region is marked with a bracket for the open complex (lane 1). Note that the open complex formation at the *Pu* promoter was blocked by the presence of the CRP-cAMP complex (compare lane 3 with lane 1).

In order to examine whether the inhibitory effect of CRP-cAMP on *Pu* operates at the transcriptional level, we detected the influence of CRP-cAMP on the open complex formation at *Pu*. The presence of open complexes may be probed by preventing transcription elongation with rifampicin to trap open complexes and then footprinting with potassium permanganate (KMnO_4_), an agent that primarily oxidizes T and C residues in single-stranded DNA of melted promoters [34]. In the presence or absence of exogenous cAMP with and without rifampicin, the *E. coli cya* mutant TP2006 containing plasmid pKU700 and pTS174 was chemically treated with KMnO_4_. The footprints obtained with the bottom strand of plasmid DNA pKU700 from intact cells are shown in Figure 2. It can be seen that in the absence of exogenous cAMP, the residues between position −9 and −5 upstream the transcriptional start site are strongly hypersensitive to attack of KMnO_4,_ indicating that open complex formed at *Pu* (Figure 2, lane 1). In contrast, in the presence of exogenous cAMP, no DNA open complexes are observed (Figure 2, lane 3), indicating that CRP-cAMP prevented XylR-dependent open complex formation at *Pu*. As negative controls, without rifampicin treating, no DNA open complexes were observed regardless of the presence or absence of exogenous cAMP (Figure 2, lanes 3 and 4). We conclude therefore, that the CRP-cAMP-mediated inhibition on *Pu* expression is at the transcriptional level.

### Overproduction of the activator alleviates the CRP-cAMP-mediated inhibitory effect on *Pu*

**Figure 3:**
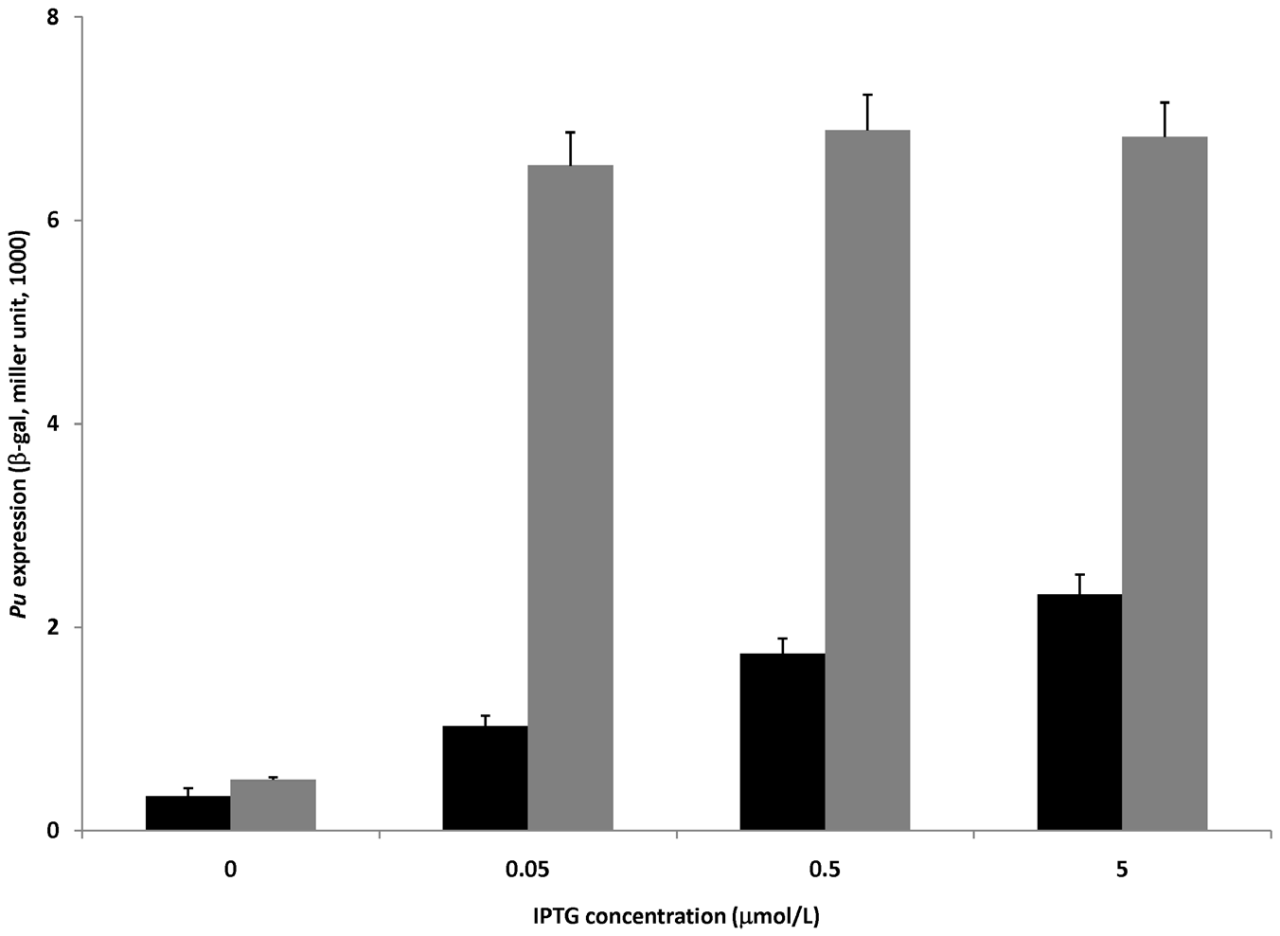
Expression of constitutively active XylR alleviates the CRP-cAMP-mediated inhibitory effect on *Pu in vivo*. *E. coli cya* mutant TP2006 was co-transformed with plasmids pKU700 + pVTRΔA (the latter carries *xylR*ΔA gene transcribed from a *Ptac/lacI^q^* system). Transformants were grown in LB medium containing 0 or 2 mmol/L exogenous cAMP and increasing concentration of IPTG (0, 0.05, 0.5, 5 µmol/L) to early to mid-log phase (OD_600_ at 0.4). Accumulation of β-galactosidase was monitored in the cultures. Gray bar, absence of exogenous cAMP; black bar, presence of exogenous cAMP (2 mmol/L).

XylR belongs to the prokaryotic enhancer binding protein family of transcriptional regulators [35], [36]. Direct interactions between activator from this family and σ^54^ or the Eσ^54^ holoenzyme have been studied [37]–[41]. We considered the possibility that CRP-cAMP may compete out XylR-Eσ^54^ interactions to prevent open complex formation at *Pu*. To examine this possibility, we took advantage of the constitutive activity of a truncated XylR derivative named XylRΔA. In XylRΔA, the N-terminal signal reception domain had been entirely deleted, but its central activation domain and the DNA binding segment remained [42]. XylRΔA is able to constitutively activate *Pu* regardless of the presence of any inducer. Plasmid pVTRΔA expresses XylRΔA from a *tac* promoter, which can be suppressed by *lacI* ^q^ encoded on the same plasmid, and induced by the presence of IPTG [43]. Consistent with previous studies, when XylRΔA (pVTRΔA) substituted for XylR, activation of the *Pu* promoter was observed (Figure 3). Moreover, as the expression of activator XylRΔA was highly induced by increasing concentrations of IPTG present in the medium, the extent of inhibition mediated by CRP-cAMP was gradually diminished (Figure 3). It thus seems that CRP-cAMP-mediated inhibition on *Pu* can be alleviated by increasing the intracellular concentration of XylRΔA (Figure 3). This observation suggests that the effects of CRP-cAMP are directly targeted to the transcription machinery by interfering with XylR-Eσ^54^ interactions.

### CRP-cAMP-mediated inhibition on the *Pu* promoter is not specific for XylR

**Figure 4:**
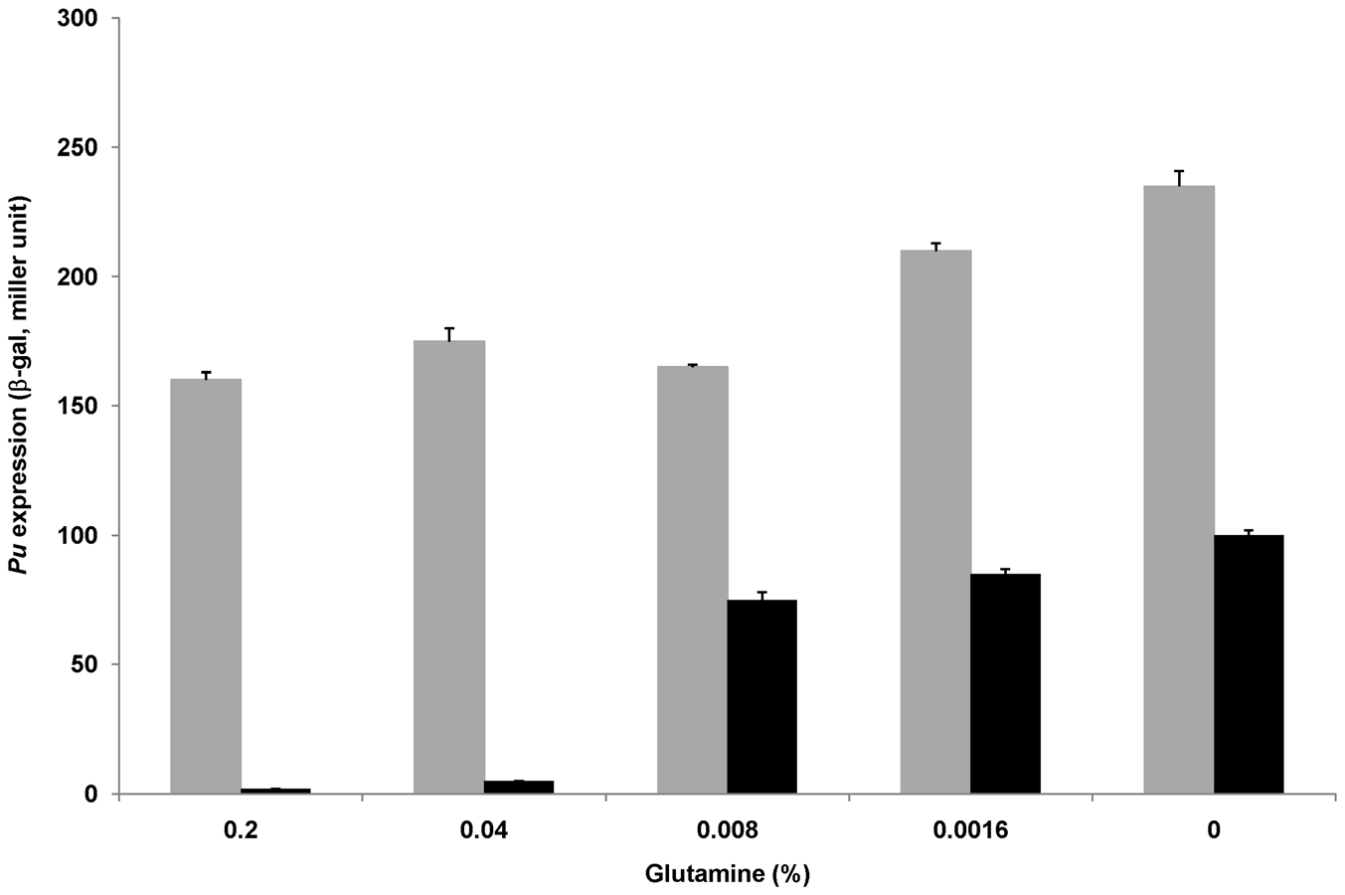
Influence of nitrogen source concentration in the medium on the *Pu* expression activated by NtrC-phosphate. In *E.coli*, *cya* mutant TP2006 strains harboring pKU700 were grown in M63 modified medium supplied with the gradually decreasing concentrations of glutamine and then measured the β-galactosidase activities. The results are the mean of at least three independent experiments and include the standard deviation. Note that when the concentration of glutamine was reduced, the extent of CRP-cAMP-mediated inhibition diminished. Gray bars, absence of exogenous cAMP; black bars, presence of exogenous cAMP (2 mmol/L).

CRP-cAMP may interact directly with XylR or with Eσ^54^ to compete out XylR-Eσ^54^ interactions. A simple possibility is that the quantity or activity of the regulatory protein XylR might be somehow modulated by the CRP-cAMP complex. To explore this and to see whether the inhibitory effect was activator-specific or not, alternative activator NtrC was used to activate *Pu* under nitrogen-deficient conditions. Under certain conditions, NtrC can activate *Pu* (as well as most other σ^54^-dependent promoters) from solution, without a need of binding to the UAS. Plasmid pKU700 was introduced into the *cya* mutant TP2006, and β-galactosidase activities were measured in the presence or absence of exogenous cAMP under nitrogen-deficient growth conditions. Activation of the *Pu* promoter was observed in the absence of CRP-cAMP, while inhibition of *Pu* was observed in the presence of CRP-cAMP. In addition, along with the reduced nitrogen supplied in the growth medium (which means increasing amount of active NtrC in the cells), CRP-cAMP-mediated inhibition on *Pu* was strongly reduced (from 50-fold down to 2-fold, Figure 4). It suggests that the increasing quantity of activator NtrC-phosphate can also lead to partial alleviation of CRP-cAMP-mediated inhibition on *Pu*, a result similar to that obtained with constitutively active XylRΔA (Figure 3). This result indicates that CRP-cAMP-mediated inhibition on *Pu* is not limited to a specific activator. Therefore, it seems improbable that CRP-cAMP specifically interferes with XylR-mediated transcriptional activation, for example by somehow interfering with the activity of XylR, e.g. by inhibiting the induced activation of XylR by mMBA. Preferably, CRP-cAMP may target to the Eσ^54^ complex at *Pu* [21], [22].

### The repression of *Pu* promoter by CRP-cAMP may occur at the step of closed complex formation

**Figure 5:**
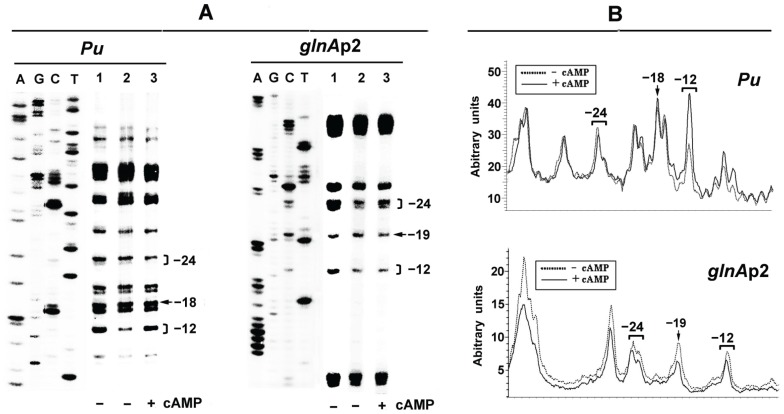
(A) *In vivo* DMS footprints to assess the interaction of Eσ^54^ with *Pu* and *glnA*p2. Plasmid pKU700 (*Pu*) or pKU101 (*glnA*p2) was footprinted in *E. coli rpoN* mutant TH1 (unable to produce σ^54^) and *cya* mutant TP2006. Lanes: 1, TH1; 2, TP2006; 3, TP2006 plus 2 mmol/L cAMP. A, G, C and T refer to sequencing lanes with the same primer. The bands in regions −12 and −24 are marked with square brackets and the reference band at −18 or −19 with an arrow. **(B) Densitometric analysis of the influence of CRP-cAMP on the interaction of Eσ^54^ with** ***Pu*** **or** ***glnA*** **p2.** The figure shows a superimposition of the normalized scans corresponding to the bands of lanes 2 and 3 in (A). The intensity of each signal is represented in arbitrary units. Note that the guanine residue within the −12 region of *Pu* becomes hypersensitive to DMS in the presence of CRP-cAMP complex.

The formation of Eσ^54^-dependent closed-complex is a rate-limiting step in the process of transcription initiation at the *Pu* promoter [11]. CRP-cAMP may inhibit transcription of the *Pu* promoter by interfering in the stable binding of Eσ^54^ to *Pu*. It was demonstrated previously that the closed-complex at the *glnA*p2 promoter can be detected by protection from dimethylsulphate (DMS) attack of critical guanines within the −12 and −24 regions of the promoter [44]. We therefore performed *in vivo* footprinting to analyze protection of the guanine residues at −14, −25 and −26 of *Pu* from DMS modification by bound Eσ^54^. In the absence or presence of 2 mmol/L exogenous cAMP, DMS footprints were conducted on plasmid pKU700 in the *cya* mutant TP2006. As controls, the *E. coli rpoN* mutant TH1 (unable to produce σ^54^) containing plasmid pKU700 was also treated with DMS. The footprints obtained with the bottom strand of plasmid DNA pKU700 from intact cells are shown in Figure 5A. It can be seen that, first, in absence of Eσ^54^, the bands at both the −12 and −24 regions of *Pu* are approximately equal in intensity to the −18 reference band (Figure 5A, Lane 1). Secondly, in the presence of Eσ^54^ and absence of CRP-cAMP, these two bands are both lower in intensity than the −18 band because the guanine residues within the −12 and −24 regions are protected by bound Eσ^54^ from methylation of DMS (Figure 5A, Lane 2). However, when CRP-cAMP is present the intensity of the band at the −12 regions was enhanced (Figure 5A, Lane 3). To obtain a more reliable reference for the influence of CRP-cAMP, the bands (Figure 5A, lanes 2 and 3) were scanned and plotted as shown in Figure 5B. Enhancement of intensity of the band at the −12 regions might reflect a conformational change of the closed complex at *Pu* promoter due to the presence of CRP-cAMP. In contrast, similar footprinting patterns were observed in the presence or absence of CRP-cAMP respectively when another σ^54^-dependent promoter *glnA*p2, which was also down-regulated by CRP-cAMP [22], [23], was used as controls (Figure 5). Therefore, the stable closed-complex formation at the *glnA*p2 promoter was hardly affected by CRP-cAMP. Taken together, the above results suggest that the mechanism by which CRP-cAMP mediated inhibition on *Pu* is different from that observed on *glnA*p2.

### AR1, but not AR2 or AR3, of CRP is essential for the inhibitory effect of CRP-cAMP on *Pu*

**Figure 6:**
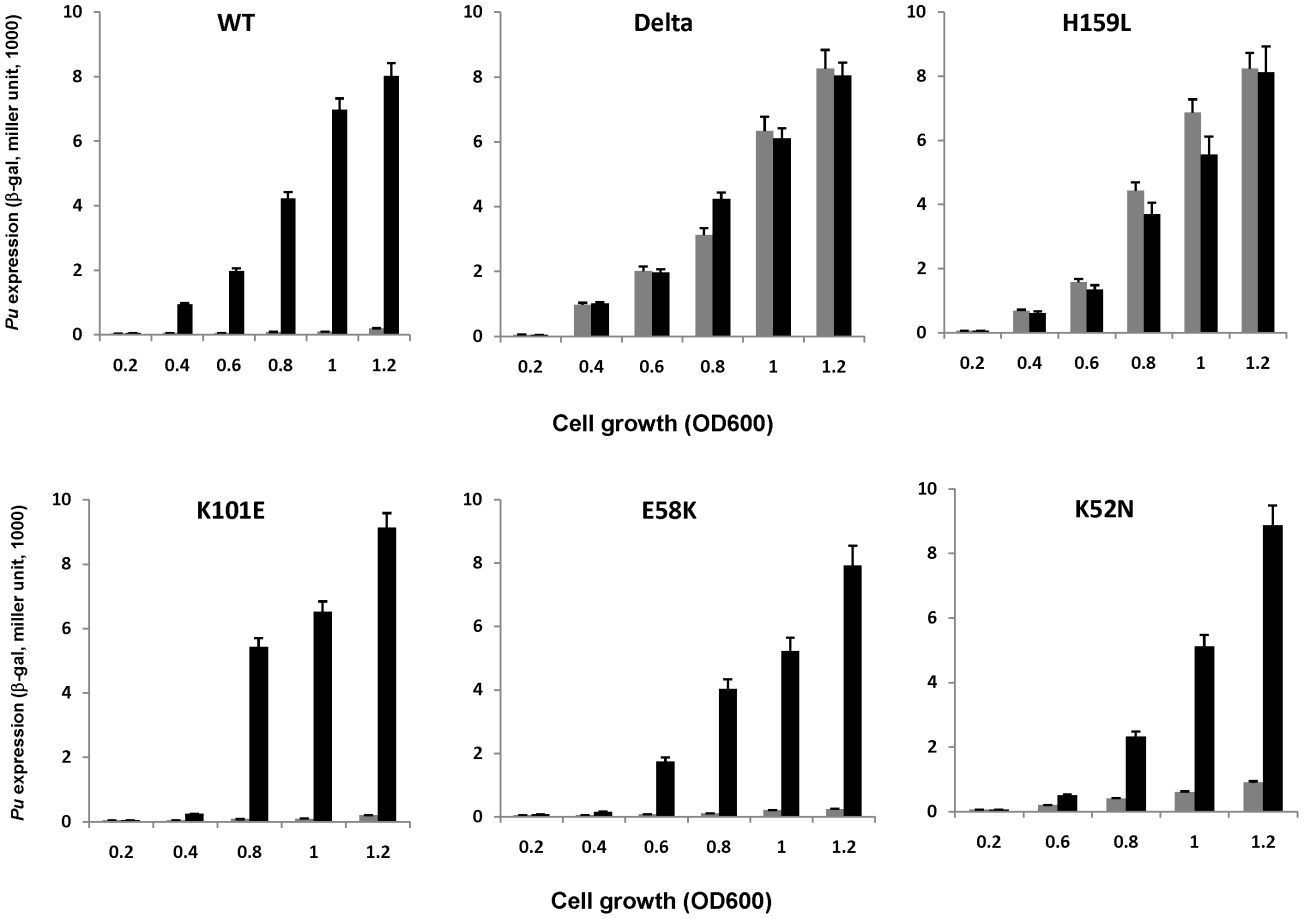
Effect of CRP mutants containing substitutions in different activating regions on *Pu* expression. A series of low copy pLG339-derived plasmids, carrying different *crp* mutants, were separately transformed into *E. coli cya crp* mutant TP2339-1 harboring pKU700 and pTS174. Accumulation of β-galactosidase was monitored during growth. Delta, representing experimental data for the pLG339ΔRS construct; black bar, absence of exogenous cAMP; gray bar, presence of exogenous cAMP (2 mmol/L).

To explore the role of the AR1, AR2 and AR3 surface determinants of CRP on *Pu*, each of these CRP mutants were tested for their ability to mediate inhibitory effect on *Pu in vivo*. Low copy pLG339-derived plasmids, harboring different mutant *crp* genes were introduced into *E. coli cya crp* double mutant TP2339-1 together with pKU700 and pTS174 respectively, using pLG339ΔRS and pLG339CRP as controls. Expression of *Pu* during growth was monitored in the presence or absence of cAMP. As shown in Figure 6, the results indicate that CRP mutants containing substitutions in AR1 lose their ability to cause inhibition on *Pu* (H159L). The same result was obtained with no *crp* gene on the plasmid (pLG339ΔRS, Figure 6). In contrast, the result obtained with the mutants in AR2 (K101E) or AR3, including both the inhibitory determinant (K52N), and activating determinant (E58K), had no significant difference from that obtained with the wild type CRP (Figure 6). These results indicated that surface AR1, but not AR2 or AR3, of CRP is indispensable for the inhibitory effect on *Pu*.

## Discussion

The *P. putida Pu* expression system has been recreated in *E. coli*. Physiological, genetic and biochemical data demonstrate that CRP inhibits *Pu* expression in a cAMP-dependent manner (Table 2). KMnO_4_ footprinting analysis indicates that the down-regulation is at the transcriptional level *in vivo* (Figure 2). The inhibitory effect of activated CRP on *Pu* remains when its cognate activator XylR is replaced by another member in the NtrC family (such as NtrC-phosphate, Figure 4). These results are in agreement with our previous observations at other σ^54^-dependent promoters [20], [22]. However, to our surprise, it was observed that such down-regulation is probably occurring through CRP-cAMP-mediated conformational change of the closed complex at *Pu* promoter *in vivo* (Figure 5). In this case, the RNA polymerase still binds the -24 region of the *Pu* promoter (Figure 5), indicating that the closed complex still exists. However, the closed complex could not be converted into an open complex efficiently in the presence of activators (Figure 2). Therefore, our results appear to indicate that the closed complex of Eσ^54^ may experience a conformational change in the presence of CRP-cAMP. Due to the complexity of the regulation of the *Pu* promoter, additional experiments may be required to fully understand the effect of CRP-cAMP on closed complex formation at the this promoter.

The finding of poised RNA polymerase in *E. coli* [45], yeast [46], mammalian embryonic stem cells [47], mammalian differentiated cells [48], and *Drosophila* [49] indicated that postrecruitment regulation occurs much more often than was previously assumed [50]. This facilitates rapid induction of the gene's expression [50]. Our observation in this study indicates that Eσ^54^ could rapidly respond the presence of PTS sugars as well as aromatic inducers to turn on the expression of certain degradation genes through a poised RNA polymerase mechanism in *E. coli*.

In this study, we find out that only the CRP mutants defective in AR1, but not in AR2 or AR3, lost the capability of inhibition on *Pu* (Figure 6), indicating that CRP-cAMP mediated inhibition relies on the direct interaction between AR1 of CRP and Eσ^54^ αCTD. AR1 of CRP is previously considered to interact with the C-terminal domain of the α subunit of RNA polymerase (αCTD) [15]. On the other hand, the recruitment of the Eσ^54^ RNA polymerase to *Pu* requires the interaction of the αCTD with the UP-like elements at *Pu* [8], [9]. Moreover, CRP can interact directly with RNA polymerase in solution [23], [51] using a method described previously [52], although there are not any potential binding sites for CRP by analyzing statistically the *Pu* promoter. The "*Pu* phenomenon" observed in this article could also be compared with what has been observed for the *dctA* and *glnA*p2 promoter. On the *dctA* promoter, binding of Eσ^54^ is not inhibited, but enhanced by the presence of CRP-cAMP *in vitro* [21]. CRP-cAMP is able to interact *in cis* from remote sites and *in trans* with the Eσ^54^-*dctA* promoter closed complex, and such an interaction prevents activator-dependent transcriptional activation [21]. On the *glnA*p2 promoter, the CRP can be recruited by Eσ^54^ to a site upstream of *glnA*p2 through the direct interaction between αCTD of Eσ^54^ and AR1 of CRP, preventing the activator protein from approaching the activator-accessible face of the promoter-bound Eσ^54^ closed complex [23]. In all cases, the Eσ^54^ in the closed complex is poised by CRP-cAMP through direct interaction between the AR1 of CRP and the αCTD of Eσ^54^.

Interestingly, when either the quantity (for the constitutive activator XylRΔA) or the activity (NtrC-phosphate) of the regulatory proteins was increased, CRP-cAMP-mediated inhibition on *Pu* was strongly reduced (from 12- and 50-fold down to 2-fold, Figure 3 and Figure 4 respectively). Since it is well known that high concentration of activator could contact the closed complex from solution without binding to UAS, the decrease of the inhibitory fold in the presence of high concentration of activator suggests that CRP-cAMP might inhibit the direct contact between the UAS bound activator and the promoter bound RNA polymerase. Many mechanisms might be involved: 1) CRP inhibits the binding of activator to UAS at *Pu* promoter (as the case of dctA, see [21]). 2) The recruitment of CRP by RNA polymerase could affect the orientation of the DNA bending between UAS and the core promoter region (as the case of *glnA*p2, see [23]). 3) The recruitment of CRP by RNA polymerase inhibits the IHF binding to its binding site.

To date, the product of *P. putida* KT2440 *crp* gene (GenBank: AE015451.1, [53], [54]) was found to have identical ARI region with CRP from *E. coli* [57]. Our data also showed that the CRP protein from *P. putida* (PpCRP) could function as a cyclic AMP receptor. When PpCRP were expressed in an *E. coli cya crp* minus strain and experiments concerning its role on the expression of *lac* promoter with exogenous cAMP were done, similar activation results were obtained from PpCRP and CRP from *E. coli* [55]. Futhermore, PpCRP was recently proved to be involved in the utilization of aromatic amino acid in cyclic AMP-dependent [56]. These observations confirmed that PpCRP was able to function as a cyclic AMP receptor in a cAMP-dependent manner. Moreover, our results showed that PpCRP could also inhibit *Pu* promoter in *E. coli* in the presence of exogenous cAMP (Table 2). Therefore, it is interesting to investigate how the activity of the equivalent *crp cya* system is controlled in its host *P. putida*.

Taken together, our data implicated that CRP can function on *Pu* promoter *in E. coli*, and maybe similarly in *P. putida*. Our results also provided proof that, despite the existence of nonspecific activator, the expression of *Pu* promoter could be inhibited by CRP in cAMP-dependent manner in *E. coli*.
